# Interleukin-35 Inhibits TNF-α-Induced Osteoclastogenesis and Promotes Apoptosis *via* Shifting the Activation From TNF Receptor-Associated Death Domain (TRADD)–TRAF2 to TRADD–Fas-Associated Death Domain by JAK1/STAT1

**DOI:** 10.3389/fimmu.2018.01417

**Published:** 2018-07-16

**Authors:** Mingzheng Peng, Yanguo Wang, Lei Qiang, Yan Xu, Cuidi Li, Tao Li, Xiaojun Zhou, Ming Xiao, Jinwu Wang

**Affiliations:** ^1^Shanghai Key Laboratory of Orthopaedic Implants, Department of Orthopaedic Surgery, Shanghai Ninth People’s Hospital, Shanghai Jiao Tong University School of Medicine, Shanghai, China; ^2^Department of Orthopedic-Spine Surgery, Binzhou Central Hospital, Binzhou Medical College, Binzhou, China; ^3^School of Biomedical Engineering, Med-X Research Institute, Shanghai Jiao Tong University, Shanghai, China; ^4^Southwest Jiaotong University College of Medicine, Chengdu, China

**Keywords:** TNF-α, osteoclast, apoptosis, FADD, interleukin-35

## Abstract

Over-activated osteoclasts derived from myeloid or peripheral blood monocytes by inflammatory cytokines results in osteoporosis, osteoarthritis, and other bone erosion-related diseases. Interleukin 35 (IL-35) is a novel anti-inflammatory and immunosuppressive factor. This study investigated the effect of IL-35 on TNF-α-induced osteoclastogenesis. In the presence of IL-35, this process was detected by Tartrate-Resistant Acid Phosphatase (TRAP) staining, F-actin staining, and bone resorption assays. The effects of IL-35 on TNF-α-induced apoptosis were demonstrated by TUNEL staining, cell viability assays, and flow cytometry. Moreover, a microarray was performed to detect the effect of IL-35 on TNF-α-activated phosphatase kinase. The effect of IL-35 on the TNF-α-mediated activation of NF-κB, MAPK, TRAF2, RIP1, Fas-associated death domain (FADD), and caspase3 was further investigated. In addition, a murine calvarial osteolysis model was established *via* the subcutaneous injection of TNF-α onto the calvaria, and histological analysis was subsequently performed. As a result, IL-35 inhibited TNF-α-induced osteoclast formation and bone resorption *in vitro* and osteolysis calvaria *in vivo*. NFATc1, c-fos, and TRAP were downregulated by IL-35 through the inhibition of NF-κB and MAPK, during which JAK1/STAT1 was activated. Moreover, based on TUNEL staining and flow cytometry, IL-35 was shown to enhance TNF-α-induced osteoclast apoptosis. Meanwhile, FADD and cleaved-caspase 3 were increased in cells treated with TNF-α and IL-35, whereas the DNA-binding activity of NF-κB was increased in TNF-α-treated cells, but was decreased in cells treated with both TNF-α and IL-35. In conclusion, IL-35 inhibits TNF-α-induced osteoclastogenesis and promotes apoptosis by activating JAK1/STAT1 and shifting activation from TNF receptor-associated death domain (TRADD)-TRAF2/RIP1-NF-κB to TRADD-FADD-caspase 3 signaling.

## Introduction

Bone is regarded as an organ that contains not only apatite or osteoid structures but also multiple cells, such as osteoclasts, osteoblasts, osteocytes, lymphocytes, macrophages, mesenchymal cells, which coordinately orchestrate the homeostasis of bone (1, 2). Specifically, osteoclasts are key mediators of bone turnover as well as certain skeletal diseases including osteoporosis, osteogenesis imperfecta, and osteoarthritis (3). Osteoclasts are one type of multinucleated cells (MNCs) that are generated from the fusion of multiple monocytes/macrophage lineages under stimulation by macrophage colony-stimulating factor (M-CSF) and receptor activator of NF-κB ligand (RANKL), which is supplied by osteoblasts and/or osteocytes.

Moreover, differentiation into osteoclasts was also found to be induced by tumor necrosis factor alpha (TNF-α), through activating of the NF-κB pathway, which particularly occurs during osteoarthritis (4). Since a large amount of TNF-α is secreted by synoviocytes and lymphocytes during osteoarthritis, subchondral bone and cartilage of joints can undergo considerable destruction by osteoclasts and proteases, including collagenase matrix metalloproteinase, which is formed in response to TNF-α stimulation (5). Secreted TNF-α within articular tissue stimulates inflammation, the production of cytokines, vascularization, and the proliferation of synoviocytes *via* NF-κB activation (6, 7). Accumulative researches demonstrated that activation of NF-κB plays a critical role in osteoclastogenesis, during which translocation of p65 into the nucleus upregulates downstream genes including c-fos and NFATc1, which are master regulators of osteoclastogenesis (8, 9).

TNF-α, produced by monocytes, macrophages, and lymphocytes, is a well-studied master pleiotropic inflammatory cytokines, attributing to the induction of many pathogenesis or diseases including osteoclastogenesis and osteoarthritis (10, 11). Forty years ago upon its initial discovery, default cellular response to TNF-α was survival and NF-κB activation (12, 13). However, recent studies have revealed that TNF-α-mediated cell death is independent of NF-κB, in which, TNF receptor-associated death domain (TRADD) and Fas-associated death domain (FADD) play critical roles (14, 15). Further studies showed that TRADD, a novel 34-kDa protein that specifically interacts with an intracellular domain of TNFR1 by death domain (DD), represents a TNFR1-associated signal transducer or bifurcation that is involved in cell death and NF-κB activation through TNFR1-TRADD-FADD-caspase3 and TNFR1-TRADD-TRAF2-IKK pathways, respectively (16). Other research also demonstrated that TRADD directly interacts with TRAF2 and FADD, signal transducers that activate NF-κB and induce apoptosis, respectively (17). Upon activation of NF-κB by TNF-α, TRAF2, a ring finger protein, recruits IκBα kinase (IKK, needed for NF-κB activation) to the TNF receptor, whereas RIP mediates IKK activation (17, 18). Moreover, TRADD also interact with FADD *via* the DD to subsequently recruit caspase-8, resulting in the formation of the death-inducing signaling complex (DISC) to active apoptosis or necroptosis (14, 19).

Interleukin 35 (IL-35), a novel member of the IL-12 family, which also includes IL-12, IL-23, and IL27 (20) is a dimeric cytokine with two subunits, specifically IL-12A and Epstein–Barr virus-induced 3 (EBI3) that are subunits of IL-12 and IL-27, respectively (21). It has been reported that IL-35, mainly secreted by regulatory T cells and B lymphocytes, elicits obvious anti-inflammatory and immunosuppressive effects (22, 23). In humans and mice, IL-35 induces the conversion of effective T cells into a new class of regulatory T cells, iTr35 cells (24). Unlike other members of the IL-12 family, IL-35 can suppress the production and proliferation of CD4+ T cells including Th1 and Th17 *via* the secretion of IL-10 from regulatory T cells. Moreover, Niedbala et al. reported that IL-35 can effectively attenuate established collagen-induced arthritis in mice, with concomitant suppression of IL-17 production but enhanced IFN-gamma synthesis (25). Recently, Jiang et al. demonstrated that IL-35 inhibited angiogenesis in arthritis, which was regarded as a novel treatment (26).

Therefore, in this present study, we assumed that IL-35 might affect TNF-α-mediated osteoclastogenesis. Specifically, it was hypothesized that IL-35 should promote apoptosis of osteoclasts *via* TNF-α associated death pathway. In the present study, we demonstrated that IL-35 inhibited TNF-α-induced osteoclastogenesis and promoted apoptosis of osteoclasts *via* the TNFR1-TRADD-FADD pathway by activating JAK1/STAT1 while suppressing NF-κB and MAPK.

## Materials and Methods

### Reagents, Samples, Cells, and Animals

Recombinant cytokines used in this study, including M-CSF, RANKL, and TNF-α, were all purchased from R&D systems (Minneapolis, MN, USA). Alpha Modification of Eagle’s Medium (α-MEM) was obtained from Hyclone (Logan, UT, USA). Penicillin–streptomycin solution, trypsin-ethylenediaminetetraacetic acid solution (0.25%), and fetal bovine serum (FBS) were obtained from Gibco (Gaithersburg, MD, USA). Cell counting kit-8 (CCK-8) was provided by Dojindo Molecular Technology Inc. (Kumamoto, Japan). The RNA extraction kit (RNeasy kit) was obtained from Qiagen (Valencia, CA, USA). Specific primary and secondary antibodies, including those targeting IkBα, P-IkBα, ERK, p-ERK, JNK, p-JNK, p38, p-p38, TNFR1, TNFR2, TRADD, FADD, cleaved-caspase8, caspase8, Stat1, p-Stat1, Stat3, p-Stat3, Stat4, p-Stat4, Jak1, p-Jak1, Jak2, p-Jak2, GAPDH, were purchased from Cell Signaling Technology (Danvers, MA, USA), Abcam (Shanghai, China) and Santa Cruz Biotechnology (Santa Cruz, CA, USA). Recombinant mouse IL-35 was purchased from sigma-aldrich.

RAW264.7 cells were provided by Shanghai Institutes for Biological Sciences, Chinese Academy of Sciences, and used as the precursor of osteoclasts. C57BL/6 mice (8 weeks old, 20–23 g) were obtained from SLAC Laboratory Animal Co., Ltd. (Shanghai, China). Animal care and experiments were conducted in accordance with guidelines and procedures authorized by the Animal Care and Use Committee of Shanghai Jiao Tong University School of Medicine. This study was carried out in accordance with the recommendations of the Institutional Review Board of Shanghai Ninth People’s Hospital with written informed consent from all subjects. The protocol was approved by the Institutional Review Board of Shanghai Ninth People’s Hospital.

### Isolation and Culture of Bone Marrow Monocytes (BMMs) and TNF-α-Induced Osteoclastogenesis *In Vitro*

Bone marrow monocytess have been regarded and used as osteoclast precursors in many studies (27–29). As previously described, primary BMMs were isolated from the bone marrow of 4- to 6-week-old C57BL/6 mice. Briefly, the femur and tibia were isolated aseptically, and epiphyses were cut off carefully after mice been euthanatized. Then, BMMs were flushed out from marrow cavity using a 1 ml syringe and incubated in α-MEM containing 10% (v/v) FBS, 100 IU/ml penicillin G, 100 g/ml streptomycin, and 30 ng/ml M-CSF at 37°C with 5% CO_2_ atmosphere overnight. Next day, media were discarded and cells were vigorously washed with PBS to remove non-adherent cells. After continuously incubated with 30 ng/ml M-CSF for 3 days, the adherent cells were regarded and used as BMMs for subsequent assays. 1 × 10^4^ cells/well of BMMs were seeded onto 96-well plates and cultured with 30 ng/ml M-CSF and 50 ng/ml TNF-α with or without IL-35 for 5 days, during which culture media were replaced every other day. Multiple nuclei giant cells (MNCs) were identified as mature osteoclasts.

### Cytotoxicity Assay

Cell viability was determined by CCK-8 assays. 1 × 10^4^ cells/well of RAW 264.7 and BMMs were seeded onto 96-well plates and incubated in complete media (100 μl/well) containing 0, 25, 50, 100, or 200 ng/ml IL-35 for 24, 48, 72, and 96 h. In addition, those cells were as well cultured in media containing 0 or 100 ng/ml IL-35 with or without 50 ng/ml TNF-α for 48 h. At each time points, and according to the manufacturer’s instructions, CCK-8 solution (10 μl/well) was added into the media and then 96-well plates were incubated in 37°C for 2 h. The absorbance was immediately detected at a wavelength of 450 nm (630 nm as reference) using a Bio-Tek Synergy HT spectrophotometer. The experiment was independently repeated three times.

### Tartrate-Resistant Acid Phosphatase (TRAP) Staining

*In vitro* osteoclastogenesis from BMMs induced by TNF-α was stained by TRAP staining solution. After culture media was discarded and cells were washed with PBS, mature osteoclasts in 96-well plates were fixed by 4% paraformaldehyde for 30 s and then covered with TRAP solution followed by incubation at 37°C for 1 h. Positive-stained MNCs (nucleus > 3) were regarded as mature osteoclasts and counted under a microscope. The average numbers of TRAP-positive MNCs were calculated from five randomly selected views under a microscope for each group. Meanwhile, the percentages of TRAP-positive staining area (per millimeter square) were measured using Image-Pro Plus 6.0 software.

### F-Actin Ring Formation Assay

F-actin rings in mature osteoclasts were stained as previously described (27). Briefly, after old media was discarded and cells were washed with PBS, cells were fixed with 4% paraformaldehyde for 10 min and then permeabilized with 0.1% Triton X-100 for 5 min before washing again with PBS. F-actin rings and nucleus were visualized using fluorescent rhodamin-conjugated phalloidin and DAPI for 10 and 5 min, respectively. Fluorescent images were captured under an LSM5 confocal microscope (Carl Zeiss, Oberkochen, Germany).

### Resorption Pit Formation Assay

1 × 10^4^ BMMs were seeded onto each bovine bone slice and incubated with complete media containing M-CSF and TNF-α in the absence or presence of IL-35 for 10 days, during which media were replaced every other day. After that, cells on the surface of the bone slice were completely removed using a trypsin-EDTA solution (0.25%) and toothbrush. Resorption pits were observed under a scanning electron microscope (SEM; FEI Quanta 250; Hillsboro, OR, USA). The number of resorption pits (per millimeter square) was counted, and the percentage of the resorption area was also measured.

### Nuclear Morphology Test

Nuclear morphology was visualized using TUNEL and Hoechst 33258 staining kit (Sigma-Aldrich, Shanghai, China) according to the manufacturer’s instruction. 1 × 10^4^ cells/well BMMs were seeded onto 96-well plate and incubated with M-CSF, M-CSF + IL-35, M-CSF + TNF-α or M-CSF + TNF-α + IL-35 for 48 h. After that, the culture solution was discarded, and 0.5 ml of fixing solution was added for 10 min. Then, the fixing solution was removed and cells were washed twice with PBS for 3 min each. 0.5 ml Hoechst 33258 staining solution was added and stained for 30 min. Blue nuclei were detected by a Fluorescence microscopy at an excitation wavelength of 350 nm and an emission wavelength 460 nm.

### Flow Cytometry

Apoptosis was detected *via* flow cytometry after staining using a FITC-Annexin V kit (BD Pharmingen, USA) according to the manufacturer’s instructions. Briefly, BMMs were pre-treated with M-CSF, M-CSF + IL-35, M-CSF + TNF-α or M-CSF + TNF-α + IL-35 for 24 h, and then collected and washed with cold PBS for three times. Then, cells were resuspended in 1 × Binding Buffer at a concentration of 1 × 10^6^ cells/ml. Then, 100 µl of the solution (1 × 10^5^ cells) was transferred into a 5 ml culture tube followed by adding 5 µl of FITC Annexin V and 5 µl PI. Cells were gently vortex and incubated for 15 min at RT (25°C) in the dark. 400 µl of 1× Binding Buffer was added to each tube before performing flow cytometry. The percentage of apoptotic cells was calculated and statistically analyzed.

### Extraction of Total mRNA and Reverse Transcription Polymerase Chain Reaction (RT-PCR)

5 × 10^5^ cells per well of BMMs were seeded onto 6-well plates and incubated for 1, 3, or 5 days. Total mRNA of cells was extracted using the Qiagen RNeasy^®^ Mini kit (Valencia, CA, USA), and then reversely transcribed into cDNA according to manufacturer’s instructions. Real-time PCR was then performed using an SYBR^®^ Premix Ex TaqTM Kit (Takara, Otsu, Japan) and an Applied Biosystems 7500 real-time PCR System. Primers of genes detected in this study were displayed in Table S1 in Supplementary Material.

### Extraction of Protein and Western Blotting

1 × 10^6^ BMMs were seeded onto 6-well plates and cultured until 90% confluence before incubation with 50 ng/ml TNF-α with or without 100 ng/ml IL-35 for 3 days. Cells were lysed in radioimmunoprecipitation assay buffer (Beyotime, Shanghai, China), pre-mixed with 1% phenylmethylsulfonyl fluoride and Phosphatase Inhibitor Cocktail (#78441; Thermo Fisher, Waltham, MA, USA) at 4°C for 30 min followed by centrifugation at 12,000 × *g* for 5 min. Supernatants were collected carefully and protein concentrations were measured using a BCA Protein Assay Reagent (Thermo Pierce, Rockford, IL, USA). Protein extracts were separated on sodium dodecyl sulfate-polyacrylamide gels and transferred onto polyvinylidene difluoride membranes (Millipore, Bedford, MA, USA). The membranes were incubated with primary antibodies at 4°C overnight, after which, secondary antibodies were added and incubated for 1 h at room temperature. Protein bands were detected using an Odyssey V3.0 image scanner (Li-COR. Inc., Lincoln, NE, USA). A Phospho-Kinase Array Kit was purchased from R&D Systems (ARY003B) and the protocol was followed as recommended by the vendor’s handbook.

### Immunofluorescence Assay

5 × 10^3^ BMMs were seeded onto dishes and incubated with media containing no FBS for starving. Two hours later, cells were stimulated with or without TNF-α, IL-35, or both TNF-α and IL-35 for 15 min. Then, cells were fixed by 4% paraformaldehyde for 30 min and permeabilized with 0.1% Triton-100. 10% BSA was used for block non-specific antigens for 1 h. Cells were incubated with primary antibodies of FADD (conjugated with Alexa Fluor^®^ 488) and NF-κB (conjugated with Alexa Fluor^®^ 647) at 4°C overnight and observed under a confocal microscope (Carl Zeiss, Oberkochen, Germany).

### Electrophoretic Mobility Shift Assay (EMSA)

5 × 10^7^ cells were seeded on 6-well plate and stimulated with or without TNF-α, IL-35, or both TNF-α and IL-35 upon reaching 90% confluence. Nuclear extracts were prepared according to the method of Schreiber et al. (30) Briefly, 6 µg of the nuclear extracts were incubated with 100 pg of ^32^P-labeled double stranded NF-κB oligonucleotide (5′-AGTTGAGGGGACTTTCCCAGGC-3′; 5′-AGTTGCCTGGGAAAGTCCCCTC-3′) in binding buffer (25 mmol/l Hepes (pH 7.9), 0.5 mmol/l EDTA, 0.5 mmol/l DTT, 1% Nonidet P-40, 5% glycerol, and 50 mmol/l NaCl) containing 2 μg of (poly)deoxyinosinic deoxycytidylic acid (poly(dI-dC)) for 30 min at room temperature. The reaction DNA–protein complexes were loaded on a native 5% polyacrylamide gel and electrophoresed at 150 V for 2 h and analyzed by autoradiography.

### siRNA Transfection

The used siRNA (5′-ACGTCATATGTGATAATGT-3′) targeting at FADD was designed and purchased from Shanghai Sangon Co., Ltd. (Shanghai, China) and transfection were performed using Lipofectamine 3000 (Thermo Fisher, Waltham, MA, USA) according to manufacturer’s instructions to transfect BMMs cells.

### Mouse Calvarial Osteolysis Model and Radiological and Histological Analysis

Thirty-two 8-week-old male mice were randomly and equally separated into four groups. The calvarial osteolysis model in mice of four groups was established by subcutaneously injecting onto calvaria with PBS, TNF-α (2 µg) alone, IL-35 (2 µg) alone, or TNF-α and IL-35, respectively on every other day for 2 weeks. On day 14, all mice were euthanized and calvaria was separated and fixed with paraformaldehyde for further analyses. Micro-CT (μCT) and X-ray were performed using a high-resolution micro-CT (μCT 80; SCANCO Medical AG, Bassersdorf, Switzerland) with an isometric resolution of 9 mm at X-ray energy settings of 70 kV, 114 mA, and 8 W.

After μ-CT imaging, the calvaria was decalcified in 10% EDTA (pH 7.4) for 4 weeks and embedded in paraffin for histological sectioning. Paraffin-embedded tissues were sectioned into 5 µm sections, mounted on super frost plus glass slides, stained with hematoxylin and eosin (H&E), Masson trichrome stain and TRAP stain, and examined by light microscopy. Immunohistochemistry was also performed. Formalin-fixed paraffin-embedded 4-µm sections were dewaxed conventionally, and endogenous peroxidases were inhibited using 3% H_2_O_2_, which was followed by blocking with BSA (Gibco; Thermo Fisher Scientific, Waltham, MA, USA). Following incubation with primary antibodies of p-Stat1, FADD, and TRAF2 (dilution, 1:100) overnight at room temperature, samples were incubated with polymerized peroxidase-labeled rabbit anti-mouse IgG (dilution, 1:100). Conventional DAB visualization was performed.

### Statistical Analysis

All results are expressed as mean ± SEM from at least three independent experiments. Statistical tests were performed using a Student’s *t*-test or analysis of variance followed by Dunnett’s test. SPSS 17.0 software (SPSS Inc., Chicago, IL, USA) was used for the statistical analysis. Statistical significance was denoted as follows: **p < 0.01 and *p < 0.05.

## Results

### Inhibitory Effect of IL-35 on TNF-α-Induced Osteoclastogenesis in BMMs *In Vitro*

**Figure 1:**
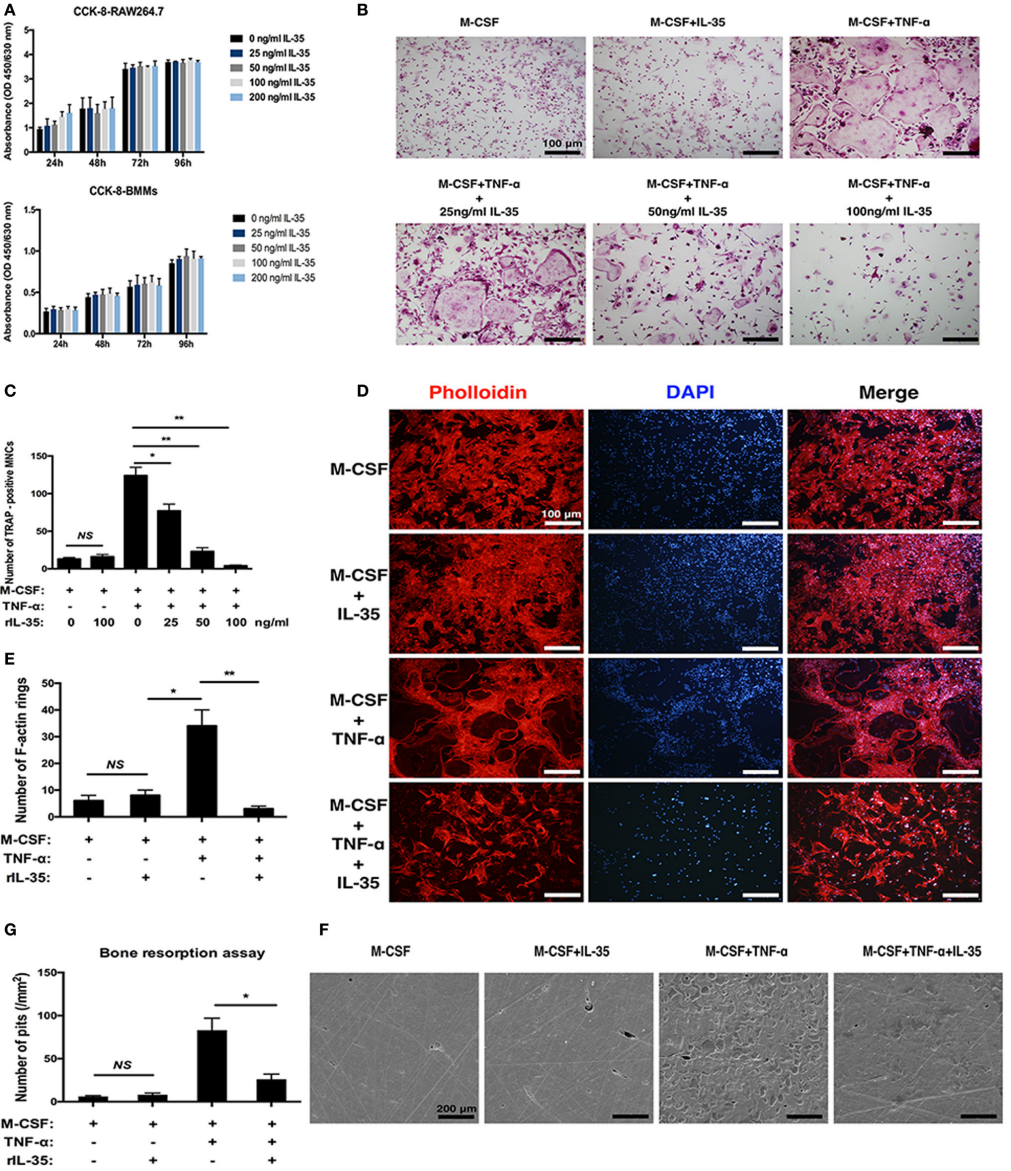
The effect of interleukin 35 on TNF-α-induced osteoclastogenesis *in vitro*. **(A)** cell viabilities were measured at the treatment of vary concentrations using Cell Count Kit-8 (CCK-8). **(B)** TNF-αN induced signaling complex and apoptotic signaling *via* TNFR1. **(C)** Cell numbers of tartrate-resistant acid phosphatase staining positive cells were counted and analyzed. **(D)** F-actin and nucleus in osteoclast were visualized by Phalloidin and DAPI, respectively. **(E)** Numbers of F-actin were counted and analyzed. **(F)** Scanning electronic microscopic images of bovine bone slices seeded with osteoclasts induced by different cytokines. **(G)** Numbers of bone resorption pits were counted and analyzed. *p < 0.05, **p < 0.01.

As is well known, TNF-α can induce osteoclastogenesis by activating NF-κB (31, 32). Therefore, in this study, we first investigated the effect of IL-35 on TNF-α-induced osteoclastogenesis *in vitro*. The IL-35 concentration used was 100 ng/ml that showed no cytotoxicity based on the consequences of cell viability assays (Figure 1A). According to TRAP staining, TNF-α markedly induced osteoclasts formation compared to that in the control group, whereas IL-35 did not make any difference without TNF-α (Figure 1B). However, IL-35 significantly inhibited TNF-α-induced osteoclastogenesis and decreased the number of mature MNCs in a dose-dependent way (*p* < 0.05, Figures 1B,C).

In addition to the formation of osteoclasts induced by TNF-α were investigated in the presence of IL-35, their function was further studied. F-actin rings are regarded as critical for the formation of seal zone and brush border of osteoclasts adjacent to bone surface for resorbing mineral matrix (33). In our research, F-actin rings were noticeably formed in TNF-α treating cells (*p* < 0.05) but significantly inhibited by IL-35 (*p* < 0.01, Figures 1D,E). Furthermore, bone resorption assays suggested the presence of numerous resorption pits in TNF-α-induced osteoclasts (*p* < 0.01). IL-35 alone (without TNF-α) did not affect the surface of bovine bone slices (*p* > 0.05, Figures 1F,G). However, IL-35 dramatically inhibited TNF-α-induced bone resorption of osteoclasts and eventually decreased the number of resorption pits (*p* < 0.05, Figures 1F,G). From these results, IL-35 suppresses TNF-α-induced osteoclast formation and function.

### IL-35 Downregulates Osteoclastogenesis-Associated Genes Including NFATc1 and c-fos

**Figure 2:**
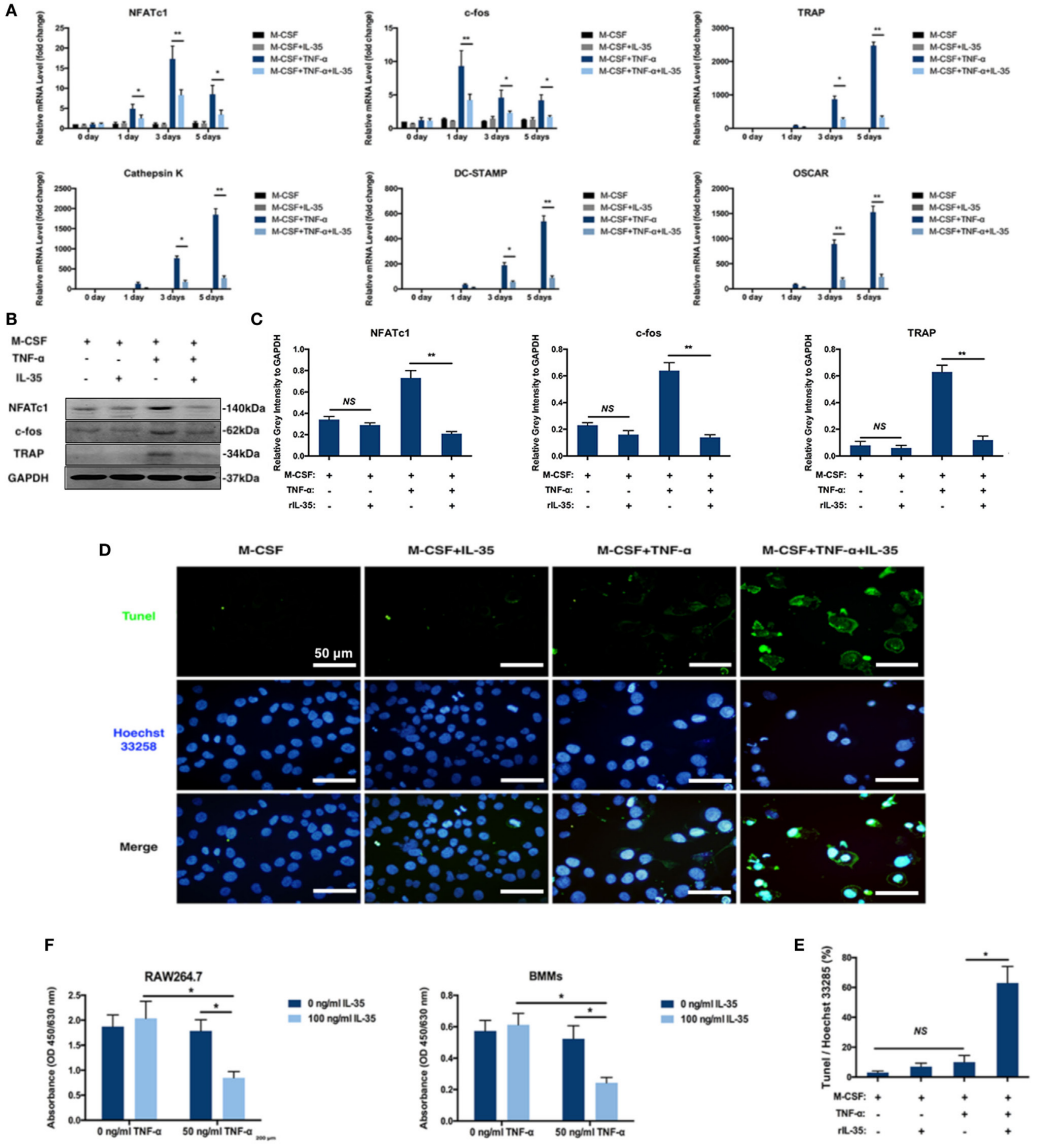
The effect of IL-35 on the expression of osteoclastogenesis associated genes and TNF-α-induced apoptosis. **(A)** RT-PCR analysis to detect the expression of osteoclastogenesis-related genes. **(B)** NFATc1, c-fos, and tartrate-resistant acid phosphatase (TRAP) were measured by western blot. **(C)** The relative gray intensity of NFATc1, c-fos, and TRAP to GAPDH. **(D,E)** Tunel stain of bone marrow monocytes treated with different agents. **(F)** Cell proliferation detected by Cell Count Kit-8 (CCK-8). *p < 0.05, **p < 0.01.

There are several related genes playing critical roles in the regulation of osteoclastogenesis including NFATc1, c-fos, TRAP, Cathepsin K, DC-STAMP, and OSCAR. Based on RT-PCR, these osteoclastogenesis-associated genes were significantly upregulated with TNF-α stimulation, compared to expression in the control group (*p* < 0.01, Figure 2A). Moreover, IL-35 greatly suppressed TNF-α-induced upregulation of NFATc1, c-fos, TRAP, Cathepsin K, DC-STAMP, and OSCAR (*p* < 0.01, Figure 2A). Accordingly, NFATc1 and c-fos proteins were detected in BMMs, and similarly, IL-35 remarkably downregulated their expression, as compared to that in the TNF-α treated group (*p* < 0.01, Figures 2B,C).

### Effect of IL-35 on TNF-α-Induced Apoptosis in Osteoclasts and Increased FasL in Bone Marrow Non-Adherent Cells

Given the fact that TNF-α induces cell death including apoptosis and necrosis in many cells and the number of BMMs treated with both TNF-α and IL-35 were dramatically decreased based on TRAP staining assays, it is reasonable to assume that IL-35 not only inhibited TNF-α-induced osteoclastogenesis but also promoted TNF-α-induced apoptosis. Therefore, apoptosis was assessed in cultured BMMs after TNF-α treatment with or without IL-35 by TUNEL and Hoechst 33258 staining. Normal cell morphologies and few TUNEL-positive cells were observed in control and IL-35-treated groups as well as in the TNF-α treated group, with similar proportions (*p* > 0.05). However, particular nuclear morphologies and significantly enhanced TUNEL staining were abundantly observed in the TNF-α and IL-35-treated group, as compared to those in the TNF-α-treated group (*p* < 0.05, Figures 2D,E). Nuclear fragmentation, visualized by Hoechst 33258 staining, was also observed in cultures treated with a combination of TNF-α and IL-35. This result suggests that IL-35 promotes TNF-α-induced apoptosis in BMMs during the process of osteoclast differentiation. Next, cell proliferation was detected using CCK-8 assays. As a result, IL-35 or TNF-α alone had no significant effect on cell proliferation compared to that in the control group. However, when cells were treated with both TNF-α and IL-35, viability was dramatically reduced (*p* < 0.05, Figure 2F).

**Figure 3:**
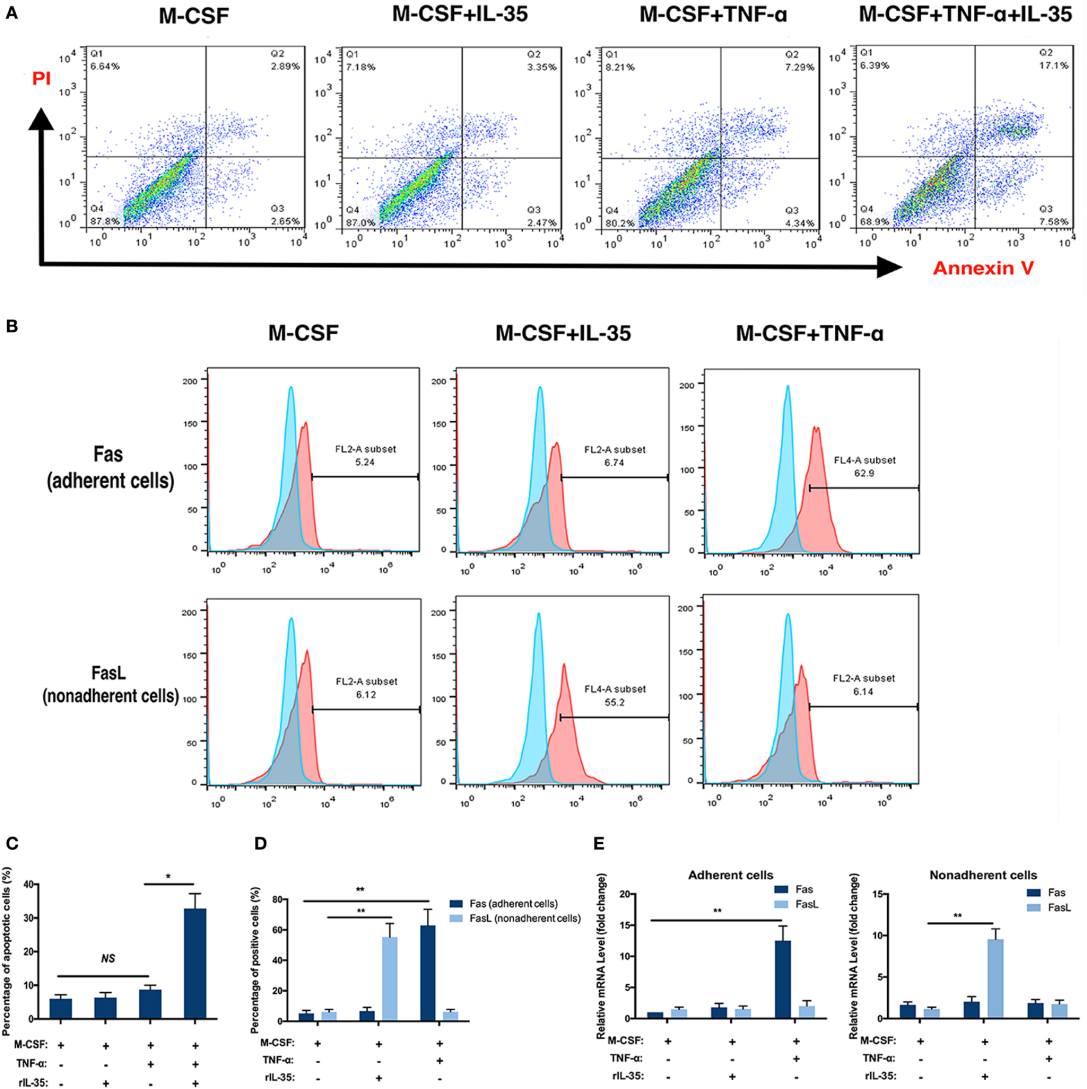
Flow cytometry analysis. **(A,C)** Apoptosis analysis using Annexin V/FITC-PI kit. **(B,E)** Fas in adherent bone marrow cells and Fas Ligand in non-adherent bone marrow cells were detected by flow cytometry. **(D)** mRNA of Fas and Fas Ligand were detected in different groups. *p < 0.05, **p < 0.01.

Moreover, the effect of IL-35 on the TNF-α-induced apoptosis of bone marrow cells was investigated using an Annexin V/FITC-PI kit and flow cytometry. After incubation with TNF-α for 24 h, 10.7 ± 3.2% of cells were apoptotic, which was not significant compared to proportions of 5.0 ± 1.2 and 5.3 ± 1.5% observed in control or IL-35 only treated groups (*p* > 0.05). In addition, when cells were incubated with IL-35 and TNF-α simultaneously, 32.8 ± 4.4% cells were apoptotic, which was significantly different compared to that in the TNF-α-treated group (p < 0.05, Figures 3A,C).

The receptors TNFR1 and TNFR2, as well as the TNF-α-induced cell death pathway, have been called death receptors or the death pathway (34). Moreover, it was previously demonstrated that TNF-α not only increases the expression of Fas on the cellular membrane of adherent bone marrow cells but also is a ligand for Fas, which is also regarded as a DR (35, 36). Therefore, the effect of IL-35 on the expression of Fas/FasL on bone marrow cells was further investigated by flow cytometry. As Fas and FasL are expressed by adherent and non-adherent bone marrow cells, respectively, we detected Fas on adherent cells and FasL on non-adherent cells with or without IL-35 or TNF-α treatment. The percentages of Fas-positive cells in control, IL-35, and TNF-α groups were 5.38 ± 1.84, 6.27 ± 2.13, and 63.40 ± 9.55%, respectively. Moreover, the percentages of FasL-positive cells in these groups were 6.05 ± 1.73, 54.87 ± 8.99, and 6.02 ± 1.68%, respectively. Therefore, it was concluded that IL-35 significantly increases expression of FasL in non-adherent bone marrow cells, whereas TNF-α increases Fas in adherent cells, which could partially account for the fact that IL-35 was shown to promote TNF-α-induced apoptosis (*p* < 0.01, Figures 3B,D). Meanwhile, mRNA levels of Fas and FasL were detected by RT-PCR, and results showed that in adherent cells, Fas was upregulated in the TNF-α treated group 12.53 ± 2.34-fold compared to expression in the control group (*p* < 0.01, Figure 3E). Moreover, in non-adherent cells, FasL was increased 9.55 ± 1.26-fold in the IL-35-treated group compared to that in the control group (*p* < 0.01, Figure 3E).

### Effect of IL-35 on TNF-α-Activated Phosphokinase Antibody Microarray Profile

**Figure 4:**
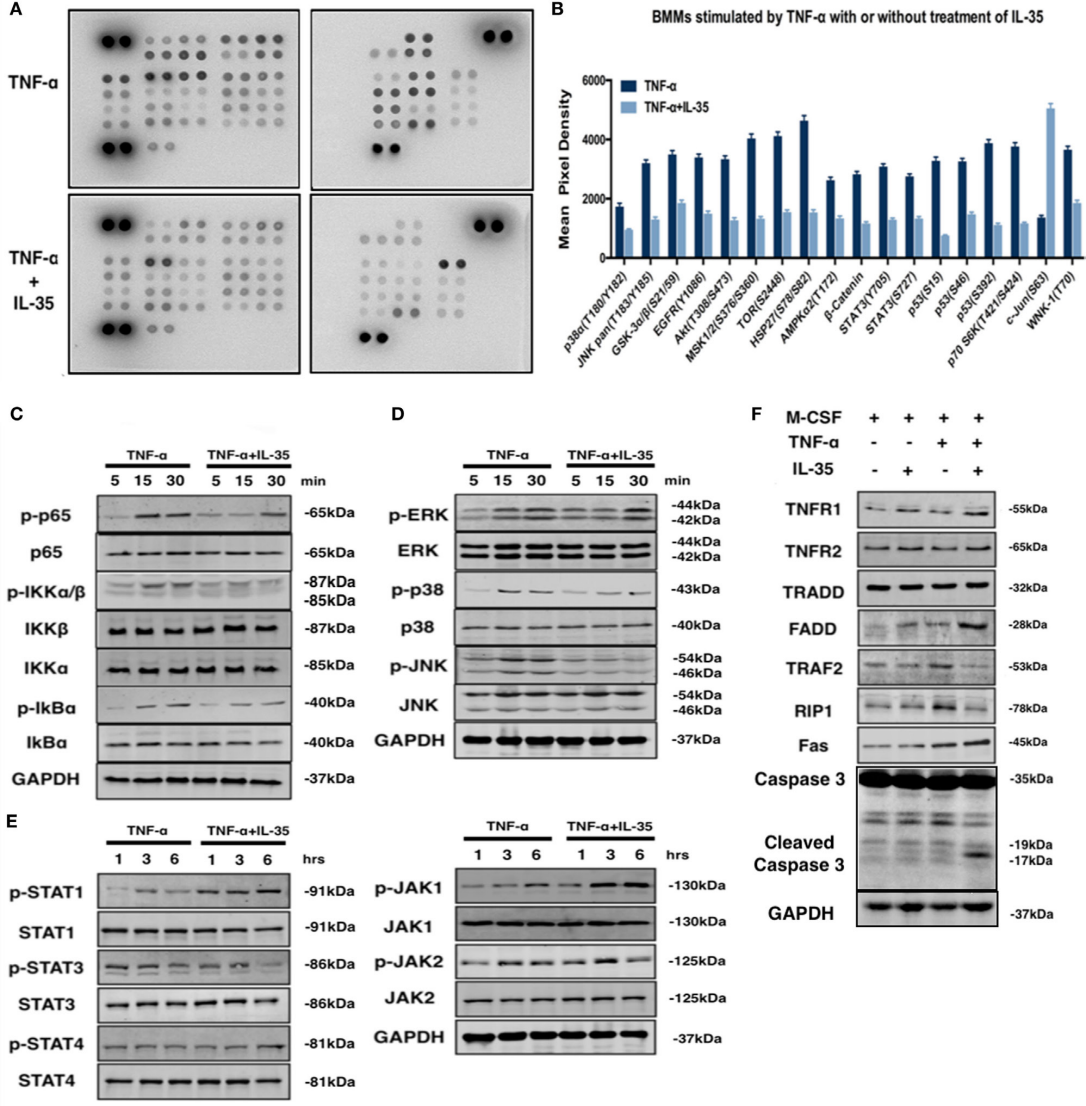

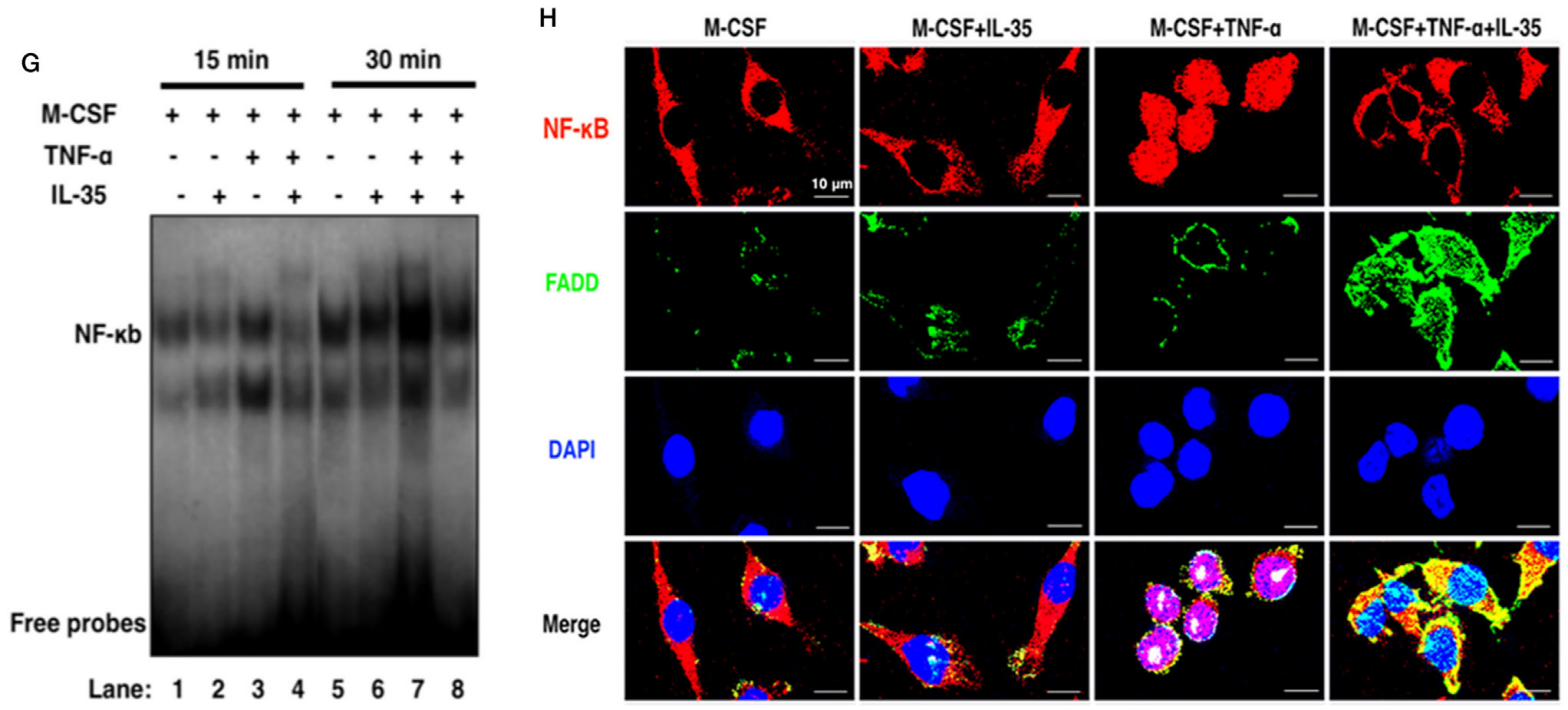
The effect of interleukin 35 (IL-35) on two pathway of TNF-α. **(A,B)** Phosphatase kinase antibody microarray (ARY003B, R&D systems) was used to analyze the effect of IL-35 on TNF-α-activated signal pathway. **(C–E)** Activation of NF-κB, MAPK, and JAK/STAT induced by TNF-α in the presence or absence of IL-35. **(F)** Molecules in TNFR1-TNF receptor-associated death domain-Fas-associated death domain (FADD)-caspase 3 pathway were detected by western blot. **(G)** Electrophoretic mobility shift assay performed to measure the DNA binding activity of NF-κg were detected in different group. **(H)** The effect of IL-35 on the expressions of p65 and FADD in bone marrow monocytess with or without TNF-α were detected by immunofluorescence staining. *p < 0.05, **p < 0.01.

To study the molecular mechanisms underlying the inhibitory effect of IL-35 on TNF-α-induced osteoclastogenesis and the enhancing effect on TNF-α-induced apoptosis, a phosphokinase array was performed using an antibody chip (Figure 4A). IL-35 significantly inhibited the phosphorylation of multiple kinases activated by TNF-α, including p38α, GSK-3α/β, Akt, AMPKα2, β-catenin, STAT3, p53, p70 s6k, and MSK, among others (*p* < 0.05, Figures 4A,B). In general, IL-35 inhibited many of the kinases activated by TNF-α.

### IL-35 Shifts Activating Effects of TNF-α From the TRADD-TRAF2-RIP1-NF-κB Survival Pathway to the TRADD-FADD-caspase8 Death Pathway *via* JAK1/STAT1

NF-κB and MAPK are predominately activated by TNF-α in many pathologic responses including immune responses and inflammation, and IL-35 has frequently been reported to suppress the immune response and inflammation (21, 37). Hence, the effect of IL-35 on TNF-α-stimulated NF-κB and MAPK was investigated. According to western blotting, IL-35 suppressed TNF-α-induced activation of NF-κB including p-p65, p-IKKα/β, and p-IkBα (Figure 4C). Meanwhile, regarding MAPKs, p-ERK, p-38, and p-JNK were also inhibited by IL-35 compared, as compared to levels in the control group (Figure 4D). As a new member of the IL-12 family, in which JAKs and STATs play pivotal functional roles, it would be reasonable to observe that phosphorylation of JAK-STAT is activated by IL-35. Consequently, it was demonstrated that p-JAK1, p-STAT1, and p-STAT4 were significantly activated by IL-35 as compared to that in control samples, whereas p-STAT3 was unexpectedly inhibited, which was consistent with the results of the antibody microarray (Figure 4E).

**Figure 5:**
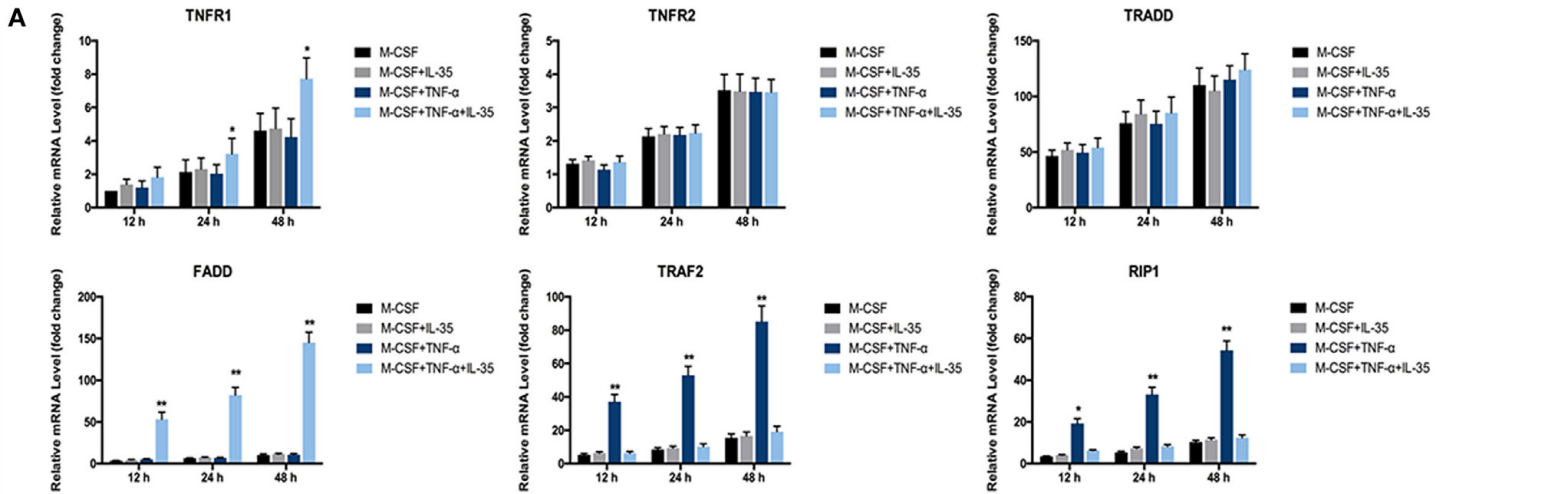

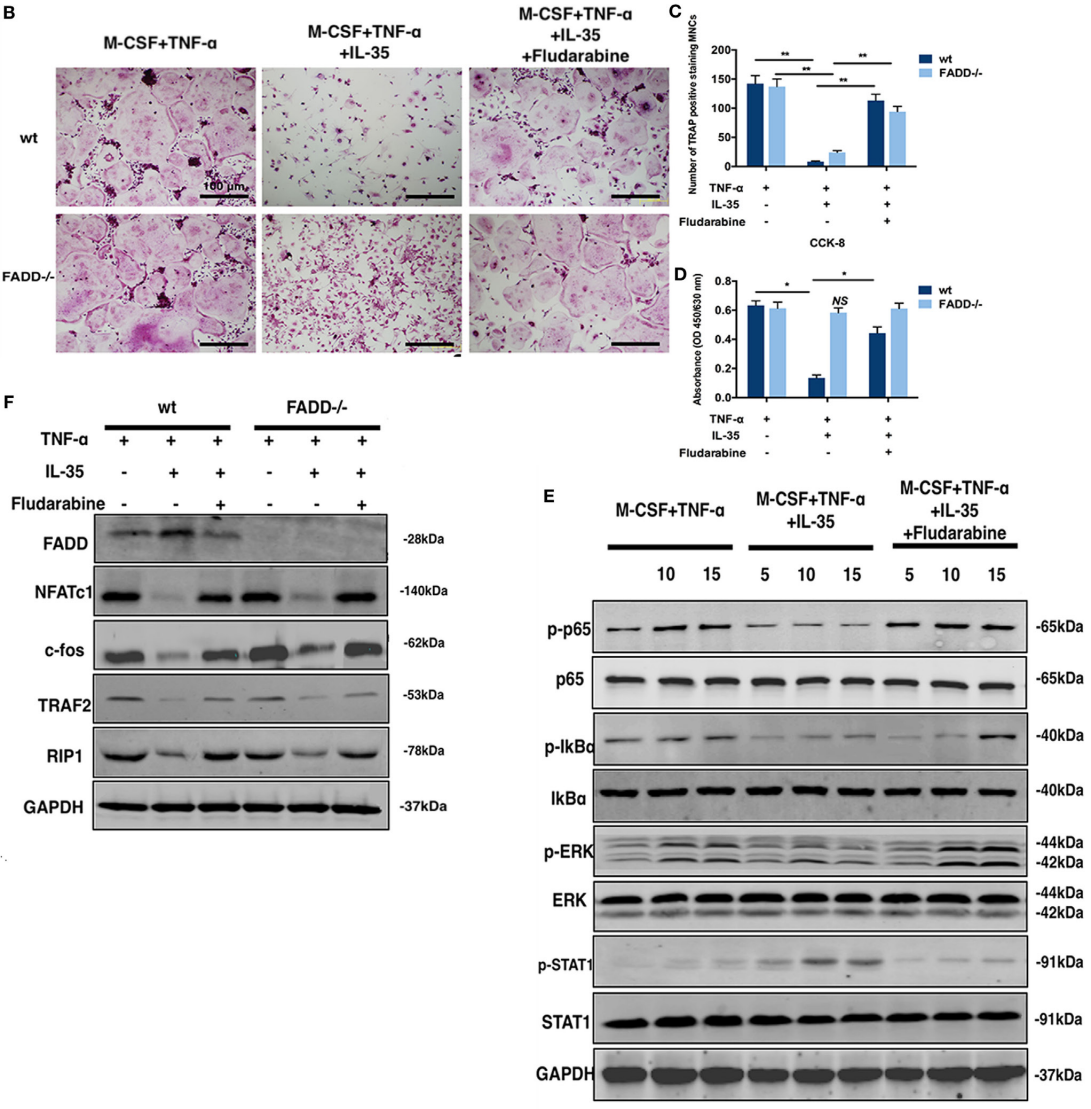
Rescuing experiments using Fludarabine as a p-STAT1 inhibitor. **(A)** mRNA of molecules in cell death pathway of TNF-α were detected by RT-PCR. **(B,C)** tartrate-resistant acid phosphatase staining of osteoclastogenesis from both wild-type and Fas-associated death domain (FADD)^−/−^ bone marrow monocytes (BMMs) induced by TNF-t, interleukin 35 (IL-35), or fludarabine. **(D)** Cell viability of wild-type and FADD^−/−^ BMMs under the stimulation of TNF-α, IL-35, or Fludarabine were detected using Cell Counting Kit-8. **(E)** Phosphorylations of NF-κB, ERK, and STAT1 in the presence or absence of Fludarabine were detected by western blot. **(F)** FADD, NFATc1, c-fos, TRAF2, and RIP1 were investigated in wild type and FADD^−/−^ cells. *p < 0.05, **p < 0.01.

A previous study showed that TNF-α can induce apoptosis and activation of NF-κB, which diverges at TRADD after ligand-induced TNFR1 trimerization. TRADD then directly interacts with TRAF2 and FADD, signal transducers that activate NF-κB and induce apoptosis, respectively (17). Therefore, the expression of these TNF-α-associated molecules was investigated. We found that TRAF2 and RIP1 were upregulated in TNF-α treated cells. However, TNFR1, FADD, and Fas were upregulated in IL-35 and TNF-α treated cells. This indicates that the TNF-α-induced death pathway was activated by IL-35. Meanwhile, TRAF2 and RIP1 in IL-35 and TNF-α treated cells were simultaneously downregulated, suggesting that the NF-κB survival pathway was inhibited at the same time (Figure 4F). RT-PCR was also performed, and as expected, it was observed that mRNA levels of TNFR1 and FADD were increased, whereas those of TRAF2 and RIP1 ware reduced in cells incubated with TNF-α and IL-35, as compared to levels in TNF-α treated cells (*p* < 0.01, Figure 5A). Subsequently, caspase 3, regarded as an executioner of TNF-α-induced apoptosis, respectively, was detected in different groups. According to the results, only in TNF-α and IL-35-treated cells caspase 3 were significantly activated, as detected by cleaved caspase 3 compared to that in controls (Figure 4F). In addition, the DNA-binding activity of NF-κB induced by TNF-α in cells treated with or without IL-35 was analyzed by EMSA. As expected, TNF-α-induced DNA binding activity of NF-κB was remarkably enhanced compared to that in control and IL-35 only treated groups. However, when cells were incubated with TNF-α and IL-35, TNF-α-enhanced DNA-binding activity was dramatically suppressed by IL-35 (Figure 4G).

To further demonstrate that IL-35 shifts TNF-α-activated signaling from TRADD-TRAF2-NF-κB to TRADD-FADD-caspase8, we detected the location of p65 and the expression of FADD simultaneously in BMMs by immunofluorescence. It was observed that p65 (red) was located in the cytoplasm in control and IL-35 only treated cells, whereas these markers translocated to the nucleus upon TNF-α stimulation. Meanwhile, the expression of FADD (green) in control, IL-35 only, and TNF-α only treated cells was low. Nevertheless, IL-35 inhibited the TNF-α-induced re-location of p65, indicating that the NF-κB pathway was inhibited. In addition, FADD was significantly upregulated in IL-35 and TNF-α treated cells as compared to expression in the other groups (Figure 4H).

### Fludarabine Rescues the JAK1–STAT1-Dependent Effect of IL-35 on TNF-α-Induced Cell Death

Previous studies have demonstrated that fludarabine is a specific inhibitor of STAT1 phosphorylation (38, 39). Therefore, 5 µM of fludarabine, which does not demonstrate cytotoxicity, was used as an inhibitor of p-STAT1 for further investigation (40). Thus, TNF-α-induced osteoclastogenesis, with or without IL-35, and TRAP staining *in vitro* were performed in the presence or absence of fludarabine. Meanwhile, siRNA was used to downregulate FADD in BMMs. According to TRAP staining results, the inhibitory effect of IL-35 on TNF-α-induced osteoclastogenesis was rescued by fludarabine in both wild-type and FADD^−/−^ BMMs. However, although the formation of osteoclasts was inhibited in both wild-type and FADD^−/−^ cells treated with TNF-α and IL-35, it was observed that the number of wild-type cells was dramatically decreased compared to the number of FADD^−/−^ cells (Figures 5B,C). Thereafter, cell viability was detected by CCK-8 assays, and it was demonstrated that downregulation of FADD rescued the enhancing effect of IL-35 on TNF-α-induced apoptosis (Figure 5D). Additionally, downregulation of NFATc1 and c-fos by IL-35 were also recovered after incubation with fludarabine (Figure 5F).

Further, the inhibitory effect of IL-35 on TNF-α-induced phosphorylation of p65, IkBα, and ERK was attenuated by fludarabine. Meanwhile, the activation of p-STAT1 was expectedly suppressed by fludarabine, as compared to that in control cells (Figure 5E). Thus, it can be concluded that IL-35 inhibits TNF-α-induced activation of NF-κB and MAKP *via* STAT1 phosphorylation. Moreover, the inhibition of TRAF2 and RIP1 by IL-35, upstream signal transducers of the TNF-α-NF-κB pathway, was rescued by fludarabine in both wild-type and FADD^−/−^ cells (Figure 5F). In addition, the expression of FADD was increased in IL-35-treated wild-type cells, as compared to that in control wild-type cells. However, these increases were not observed in FADD^−/−^ cells. Meanwhile, the upregulation of FADD by IL-35 in the presence of TNF-α was correspondingly attenuated when p-STAT1 was blocked by fludarabine in wild-type cells (Figure 5F). Collectively, inhibition of p-STAT1 by fludarabine inhibited the enhancing effects of IL-35 on TNF-α-induced apoptosis and rescued the inhibitory effects of IL-35 on TNF-α-induced activation of NF-κB.

### IL-35 Suppresses TNF-α-Induced Osteoclastogenesis in a Mouse Model of Calvaria Osteolysis

**Figure 6:**
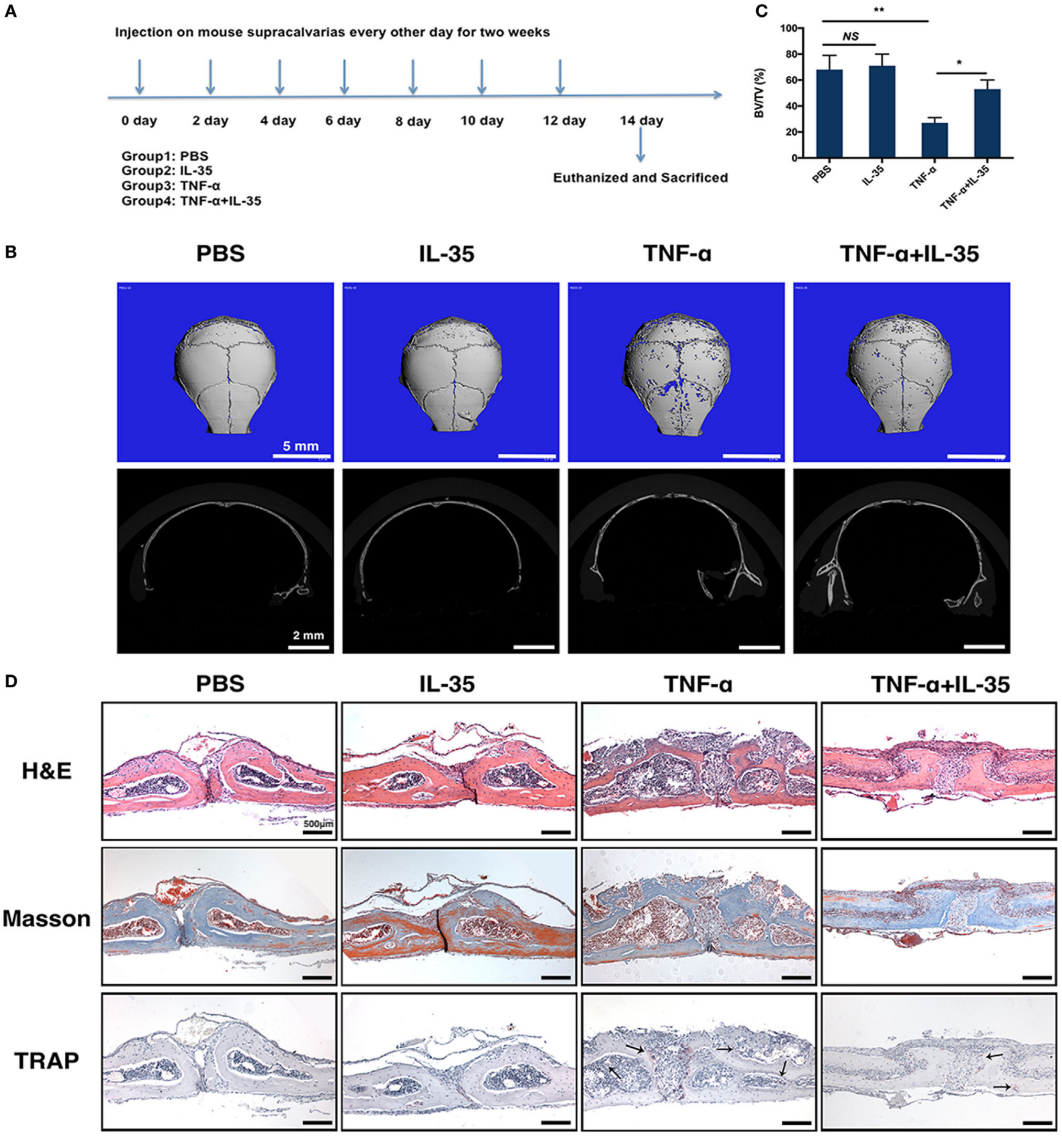
TNF-α-induced calvarias osteolysis model was established to investigate the effect of Interleukin 35 (IL-35) *in vivo*. **(A)** Timeline of animal experiments and group design. **(B)** Micro-CT analysis of calvarias injected with PBS, IL-35, TNF-α, or IL-35 and TNF-α. **(C)** Bone volume/total volume (BV/TV) were calculated and analyzed. **(D)** Hematoxylin and eosin, masson trichrome, and tartrate-resistant acid phosphatase staining of the tissue section. *p < 0.05, **p < 0.01.

A mouse osteolysis model has been used to study osteoimmunology or, specifically, TNF-α-induced osteoclastogenesis (36). Therefore, we established a TNF-α-induced mouse calvaria osteolysis model to further investigate the effect of IL-35 *in vivo*. The timeline showing specific stages of the animal experiment is illustrated in Figure 6A. After subcutaneously injecting calvaria with PBS, IL-35, TNF-α, or both IL-35 and TNF-α, every other day for 2 weeks, all mice were sacrificed and calvaria was analyzed. According to μ-CT results, calvaria in the TNF-α-injected group showed significantly more bone resorption pits compared to that in the PBS group; moreover, bone volume/total volume in the TNF-α-injected group was dramatically lower than that in control animals (*p* < 0.01, Figures 6B,C). Meanwhile, IL-35 injection did not affect bone mineral density. Moreover, co-injection of IL-35 and TNF-α rescued TNF-α-mediated osteolysis (*p* < 0.05, Figures 6B,C).

Subsequently, a dramatic increase in the destruction of bone tissue and a number of inflammatory cells was observed in the calvaria of TNF-α-injected mice, as compared to those in PBS- or IL-35-injected animals, as determined by H&E and Masson trichrome staining (Figure 6D). In addition, the formation of osteoclasts *in vivo* was also detected by TRAP staining, and it was found that TRAP-positive MNCs increased in TNF-α-injected calvaria compared to that in PBS- and IL-35-injected groups. However, IL-35 co-injection inhibited the increase in TRAP-positive MNCs caused by TNF-α.

### IL-35 Increases TNF-α-Induced Osteoclast Apoptosis by Downregulating TRAF2, Upregulating FADD, and Activating p-STAT1 *In Vivo*

**Figure 7:**
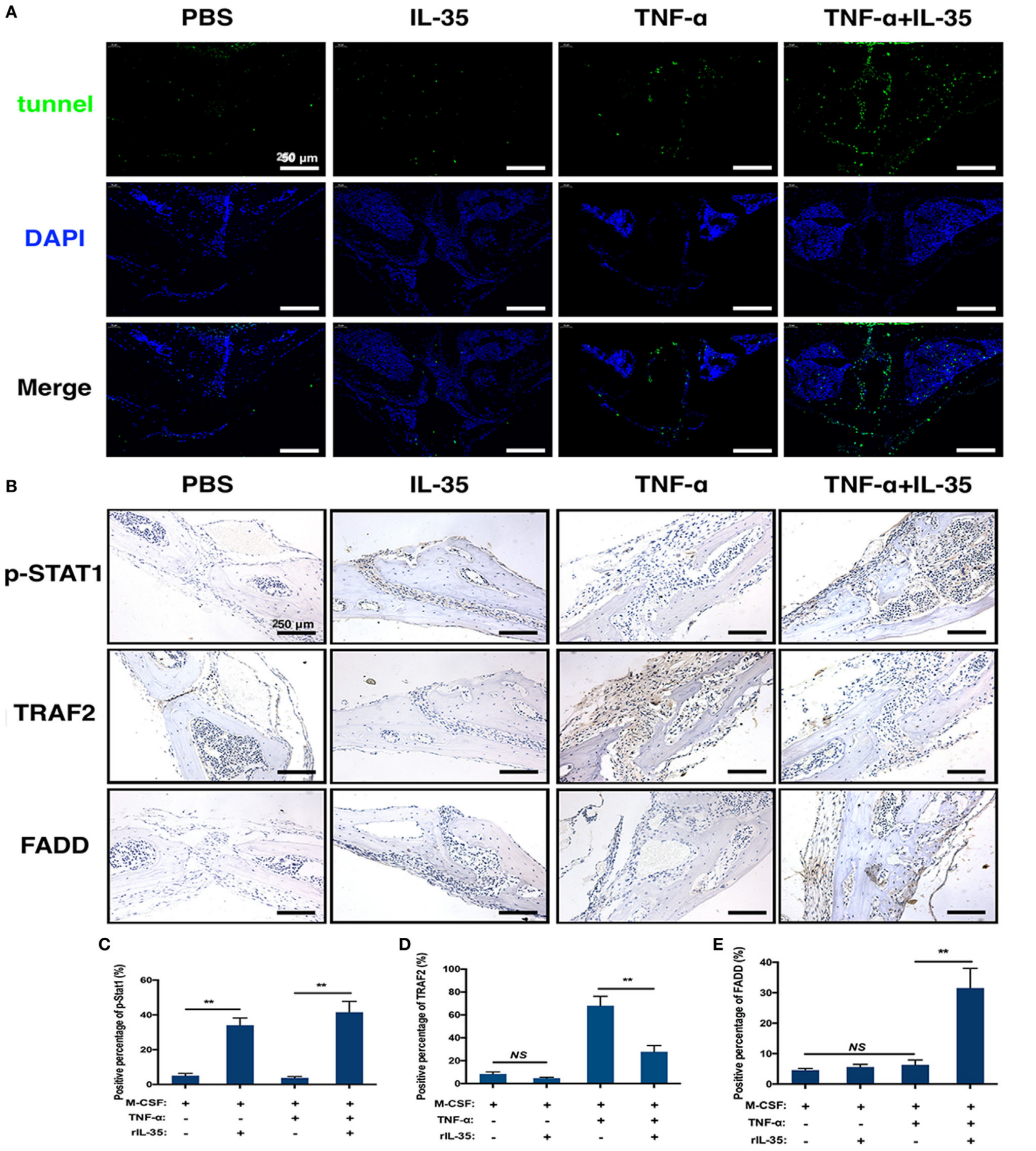
Tunel stain and immunohistochemistry analysis. **(A)** Tunel stain was performed to detect the apoptotic level in the section of calvarias from different groups. **(B–E)** p-STAT1, TRAF2, and Fas-associated death domain were detected by immunohistochemistry assay and analyzed. *p < 0.05, **p < 0.01.

TNF-α-induced apoptosis or cell death was detected by TUNEL staining in calvaria. TUNEL expression in TNF-α and IL-35 co-injected mice was significantly increased compared to that in PBS, IL-35, or TNF-d groups (Figure 7A). From this, it can be concluded that IL-35 promotes TNF-α-induced apoptosis. Furthermore, we investigated the effect of IL-35 on the two aforementioned TNF-α-associated transduction pathways *in vivo*. Based on immunohistochemistry, it was demonstrated that p-STAT1 was markedly activated in IL-35-injected mice and animals co-injected with IL-35 and TNF-α, as compared to that in the PBS group (*p* < 0.01, Figures 7B,C). Moreover, TARF2, an upstream transducer the TNF-α-induced NF-κB pathway, was highly expressed in the calvaria of mice injected with TNF-α, as compared to expression in PBS group, but this increase in expression was inhibited when IL-35 and TNF-α were co-injected (*p* < 0.01, Figures 7B,D). Meanwhile, higher FADD expression was observed only in mice co-injected with IL-35 and TNF-α (*p* < 0.01), as compared to that in the other three groups, and there was no significant difference in terms of FADD expression (*p* > 0.05, Figures 7B,E).

## Discussion

Studies have demonstrated that TNF-α is the most critical cytokines driving inflammatory responses and can be used as a target for the treatment of inflammatory diseases. However, although it was initially discovered 40 years ago, TNF-mediated cell death remains a phenomenon *in vitro*, and the underlying physiological and pathophysiological mechanisms are uncertain (12, 13). Engagement of TNFR1 and TNF-α results in the assembly of a membrane-bound primary signaling complex (complex I) that drives the activation of downstream signal transducers, which sequentially activates the formation of a secondary cytoplasmic complex (complex II) that mediates cell death (17, 41). Nevertheless, in most cells, the default pathway after activating TNFR1 signaling is the canonical NF-κB pro-survival pathway rather than the pro-cell death pathway. Therefore, although TNF-α can induce cell death, this death pathway is inhibited unless certain cell death checkpoints are destroyed eliminated.

The IL-12 family includes IL-12, IL-23, IL-27, and IL-35. According to previous research, IL-12 was shown to have the potential to inhibit osteoclast formation in mouse bone marrow cells treated with M-CSF and RANKL or TNF-α (42). Moreover, IL-12 promotes apoptosis in bone marrow cells through an interaction between Fas and Fas ligand to suppress osteoclastogenesis (43–45). However, IL-23, consisting of IL-23 p19 and IL-12 p40, promotes osteoclast formation by upregulating receptor activator of NF-κB (RANK) expression in myeloid precursor cells (46). Other research revealed that IL-23 and IL-27 can partly inhibit RANKL-induced osteoclastogenesis from BMMs without affecting their proliferation (47). Thereafter, Furukawa et al. found that IL-27 abrogates RANKL-mediated osteoclastogenesis in human granulocyte-macrophage colony-forming unit cells through the STAT1-dependent inhibition of c-fos. Therefore, as a novel member of the IL-12 family, the effect of IL-35 on osteoclastogenesis was tremendously intriguing.

First, we found that IL-35 dramatically suppresses TNF-α-induced osteoclastogenesis *in vitro*, based on TRAP staining, in a dose-dependent manner. Next, the effect of IL-35 on the function of osteoclasts was investigated, and it was demonstrated that F-actin is reduced in IL-35-treated osteoclasts induced by TNF-α. Bone resorption pit assays revealed that the formation of osteoclasts was inhibited by IL-35; bone resorption by osteoclasts was also suppressed accordingly (Figure 1). Furthermore, NFATc1, c-fos, TRAP, Cathepsin K, DC-STAMP, and OSCAR were also downregulated during IL-35-mediated inhibition of TNF-α-induced osteoclastogenesis.

In addition, it was also noted that BMM populations were dramatically reduced in the IL-35 and TNF-α co-treated group during osteoclastogenesis. Therefore, we hypothesized that IL-35 might redirect TNF-α activation from NF-κB to apoptosis during the inhibition of TNF-α-induced osteoclastogenesis. Therefore, cell viability assays were performed and cell proliferation was remarkably suppressed in the group co-treated with IL-35 and TNF-α, as compared to that in the other groups (Figure 2E). To further confirm this, TUNEL staining (Figures 2D,F) and flow cytometry using an Annexin V/FITC-PI apoptosis kit were performed (Figures 3A,C). In line with previous results, IL-35 indeed dramatically promoted TNF-α-induced apoptosis (Figure 2). Likewise, Long et al. reported that IL-35 overexpression increases sensitivity to apoptosis and suppresses cell growth in human cancer cells, resulting in cell cycle arrest at the G1 phase (48). As IL-12 was demonstrated to induce the expression of FasL on the non-adherent bone marrow cells to promote the apoptosis of osteoclasts (43), the effect of IL-35 on FasL of non-adherent cells was also investigated by flow cytometry. IL-35 significantly induced the upregulation of FasL on non-adherent cells, which interacts with TNF-α-induced Fas on adherent cells to promote bone marrow cell apoptosis.

Moreover, the mechanisms associated with the effect of IL-35 on the TNF-α-induced activation were studied. A phosphatase kinase antibody microarray was used to screen the inhibitory mechanism of IL-35. It was found that TNF-α-activated phosphorylation of p38α, GSK-3α/β, Akt, AMPKα2, β-catenin, STAT3, p53, p70 s6k, and MSK was inhibited by IL-35 (Figures 4A,B). Subsequently, we also found that IL-35 remarkably inhibited TNF-α-mediated activation of phosphatases including p-p65, p-IKK α/β, p-IkBα, p-p38, and p-JNK (Figures 4C,D). Meanwhile, p-JAK1, p-STAT1, and p-STAT4 were activated by IL-35 (Figure 4E). Hu et al. revealed that IL-35 pretreatment alleviates lipopolysaccharide-induced acute kidney injury in mice by inhibiting NF-κB activation, including expression of p-p65 and p-IKKα (49). Sha et al. reported that IL-35 inhibits endothelial cell activation by suppressing the MAPK-AP-1 pathway (50).

Thereafter, we investigated the effect of IL-35 on the TNFR1-TRADD-FADD pathway and found that TNFR1, FADD, and cleaved caspase3 were upregulated in cells co-treated with TNF-α and IL-35. In addition, the increases of TRAF2 and RIP1 induced by TNF-α as well as the DNA-binding activity of NF-κB were attenuated by IL-35 (Figure 4F). Immunofluorescence assay results also showed that IL-35 suppresses the translocation of NF-κB from the cytoplasm to the nucleus upon TNF-α activation and increases the expression of FADD (Figure 4G). Furthermore, the inhibitory effect of IL-35 on TNF-α-induced osteoclastogenesis and the enhancing effect on apoptosis were significantly rescued by fludarabine, a specific inhibitor of p-STAT1 (Figure 5). Thus, in conclusion, IL-35 inhibits TNF-α-induced activation of NF-κB and activates the FADD-caspase3 apoptosis pathway *via* JAK1/STAT1. Wang et al. previously reported that STAT1 can directly interact with TNFR1 and TRADD, as a component of the TNF-α receptor 1-TRADD signaling complex, to inhibit NF-κB activation (51). Jiang et al. recently reported that the phosphorylation of STAT1 enhanced its binding to TRADD and thus recruits FADD and caspase 8 to form DISC complexes, resulting in apoptosis but not NF-κB activation (52).

Additionally, the mouse calvaria osteolysis model was established through the injection of TNF-α to investigate the effect of IL-35 *in vivo*. In line with *in vitro* results, μ-CT measurements showed that TNF-α injection successfully induced osteolysis and obvious bone resorption pits. However, injection of IL-35 inhibited TNF-α-induced osteolysis (Figures 6B,C). According to histological analysis, it was observed that the dramatic increase in bone tissue destruction induced by TNF-α was rescued by IL-35 (Figure 6D). More TUNEL-positive cells were observed in the calvarias of mice co-injected with TNF-α and IL-35, compared to that in PBS, IL-35, and TNF-α groups, among which no significant difference was found. According to immunohistochemistry analysis, IL-35 activated p-STAT1 to increase the expression of FADD in the presence of TNF-α, but also downregulated TRAF2 (Figure 7).

**Figure 8:**
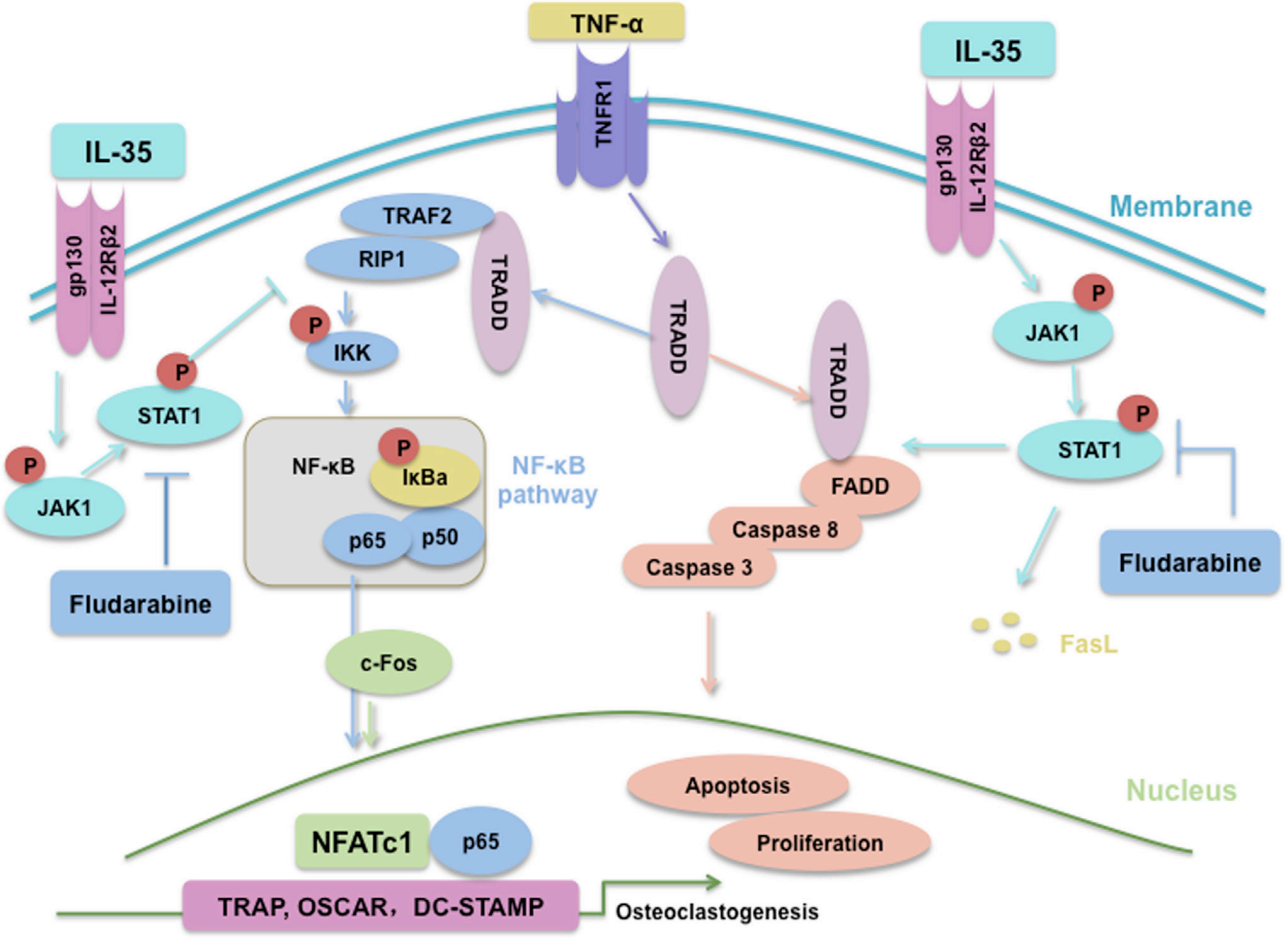
Schematic of interleukin 35 shifting the activating pathway of TNF-α from TNF receptor-associated death domain (TRADD)-TARF2/RIP1-NF-κB to TRADD-Fas-associated death domain-caspase3 to promote apoptosis of osteoclasts.

In summary, IL-35, an anti-inflammatory cytokine, inhibits TNF-α-induced osteoclastogenesis by inhibiting TRADD-TRAF2-NF-kB signaling, but promoting activation of the TRADD-FADD-caspase3 pathway, through JAK1/STAT1 (Figure 8). Based on our research, IL-35 could represent a novel treatment modality for many inflammatory diseases or cancer, based on its anti-TNF-α effects. Undoubtedly, from the perspective of translational medicine, it will make more difference if the effect of IL-35 on osteoclast of human could be investigated, which should and would be further performed.

## Ethics Statement

This study was carried out in accordance with the recommendations of guidelines and procedures authorized by the Animal Care and Use Committee of Shanghai Jiao Tong University School of Medicine. The protocol was approved by the Institutional Review Board of Shanghai Ninth People’s Hospital.

## Author Contributions

MP and JW initially designed the whole study. MP, YW, LQ, YX, and CL performed cellular experiments including cell culture, RT-PCR, western blot, and immunofluorescence assay. Also, MP, YW, TL, and XZ performed animal experiments. MP and MX analyzed all data. Eventually, MP wrote and revised the manuscript under the instructions of JW.

## Conflict of Interest Statement

The authors declare that the research was conducted in the absence of any commercial or financial relationships that could be construed as a potential conflict of interest.
