# Supplementary material for: Interleukin-35 Inhibits TNF-α-Induced Osteoclastogenesis and Promotes Apoptosis via Shifting the Activation From TNF Receptor-Associated Death Domain (TRADD)–TRAF2 to TRADD–Fas-Associated Death Domain by JAK1/STAT1
Source: Front Immunol. 2018 Jul 16;9:1417. doi: 10.3389/fimmu.2018.01417 (PMC6054960; doi:10.3389/fimmu.2018.01417)
Supplement: Supplementary file 1 [file table_1.docx]

| **Genes** | **Forward sequence** | **Reverse sequence** |
| --- | --- | --- |
| NFATc1 | CTTCCTGCCAATGTTCCAAT | TGGTGAGCTGTTGGCTGTAG |
| c-fos | CCTGGATTTGACTGGAGGTC | TTGCTGATGCTCTTGACTGG |
| TRAP | CAGCAGCCAAGGAGGACTAC | GGCTAACAATGGTCGCAAGT |
| Cathepsin K | TCTCTCGGCGTTTAATTTGG | GGTCATGTCTCCCAAGTGGT |
| DC-STAMP | TGAAAAGGAGAGGCACCAAC | AGCCCAAGGGTTTTCTTCTC |
| OSCAR | TTTTGCACTGTACCGCGTAG | AGCAACAGTAGGTGCCAGGT |
| Fas | ATGCACACTCTGCGATGAAG | CAGTGTTCACAGCCAGGAGA |
| FasL | CATCACAACCACTCCCACTG | TCCTAATCCCATTCCAACCA |
| TNFR1 | GACCGGGAGAAGAGGGATAG | TCGGACAGTCACTCACCAAG |
| TNFR2 | CTCTAAGTGCCATCCCAAGG | ACCCAACGATGTAAGGATGC |
| TRADD | GAAGTTCCCGGTTTCCTCTC | CAAACGTCTGCTGGTCTTGA |
| FADD | AGAGTGGCCTGGACCTGTTC | GCGCTGCAGTAGATCGTGTC |
| TRAF2 | CATTGTCTGCGTCTTGAACC | CCAGCTGTTGCACCTTGTTA |
| RIP1 | CCAAGGCAGAATGAGGCTTA | GACCTCTTGCTCCTGCACTC |
| GAODH | GGGTGTGAACCACGAGAAAT | CCTTCCACAATGCCAAAGTT |

Supplementary Table 1. The premier sequences of osteoclastogenesis associated genes and TNF-α related genes
